# RNA therapeutics: A promising therapy for future cardiology

**DOI:** 10.34172/jcvtr.026.33867

**Published:** 2026-03-30

**Authors:** Samad Ghaffari, Taher Entezari-Maleki

**Affiliations:** Cardiovascular Research Center, Tabriz University of Medical Sciences, Tabriz, Iran

## Introduction

 RNA therapeutics are a novel promising therapeutics with well-studied in infectious and cancer treatment. The prominent clinical role of RNA therapeutics has been pronounced by emerging mRNA-Based COVID-19 vaccines after the pandemic by a successful global use. In cardiology, RNA therapeutics are also well studied in management of hypertension, dyslipidemia, and cardiac amyloidosis and are under investigating in the treatment of other conditions such as revascularization and cardiac regeneration.^1^

 Several types of RNA therapeutics have been used or under investigating in the clinical studies. Some important types of RNA therapeutics that have been widely studied in cardiovascular disease (CVD), are as follows: Short-interfering RNA (siRNA) that degrades mRNA and inhibit subsequent protein synthesis. MicroRNAs (miRNAs) that slice mRNA and inhibit mRNA translation. Antisense oligonucleotides as single-stranded RNA, that induce mRNA destruction and change protein function by modifying mRNA merging. ^1^ Below, the clinical use of RNA therapeutics in cardiovascular therapy is discussed.

## Hypertension

 Zilebesiran is a siRNA therapeutic that specifically targets angiotensinogen (AGT) synthesis in the liver and has been investigated in the pharmacotherapy of hypertension.^1,2^

 In a phase 1 study, 107 hypertensive individuals were randomized to receive either a single subcutaneous dose of zilebesiran (10, 25, 50, 100, 200, 400, or 800 mg) or placebo with a 24-week follow-up. The results showed that a single subcutaneous dose of zilebesiran of 200 mg or more resulted in a dose-dependent decrease in serum angiotensinogen levels and 24-hour ambulatory blood pressure, which were continued for up to 24 weeks. Mild injection-site reactions were reported as adverse events.^2^

 Zilbesiran is also being investigated in KARDIA-1 and KARDIA-2 trials in individuals with mild-to-moderate hypertension without antihypertensive medications, and individuals with inadequate blood pressure control, receiving standard anti-hypertensive medications, respectively.^1,2^ Despite significant treatment success, some concerns remain over AGT elimination by Zilbesiran; where, in certain clinical situations like hypovolemia and acute kidney injury, AGT is needed in the management of these clinical scenarios. Further safety profile studies are also required to determine the zilebesiran drug interaction with other medications, as well as its cost-effectiveness compared to other antihypertensive medications.^1,2^

## Hypercholesterolemia and Dyslipidemia

 Several siRNA therapies are under consideration for the treatment of hypercholesterolemia and dyslipidemia. Among them, inclisiran, a siRNA therapeutic, received FDA approval for the treatment of atherosclerotic cardiovascular disease and heterozygous familial hypercholesterolemia by targeting proprotein convertase subtilisin/kexin type 9 (PCSK9), which promotes low-density lipoprotein (LDL) receptor degradation.^1^

 In the phase-2 ORION-1 trial on 507 patients, subcutaneous injection of 300 mg inclisiran delivered on days 1 and 90 showed the greatest reduction in LDL cholesterol levels, with 48% of the patients showing LDL-C levels lower than 50 mg per deciliter at day 180.^3^

 In phase 3 trials of ORION-10 and 11, inclisiran showed a ≈50% reduction in LDL levels at day 510 of the trial among patients with atherosclerotic cardiovascular disease with elevated LDL-C despite receiving a maximum tolerated statin in all cohorts.^4^

 Plozasiran (ARO-APOC3) is a siRNA that targets APOC3 (apolipoprotein C-III), recently approved as an adjunct to diet to reduce triglycerides in adults with familial chylomicronemia syndrome (FCS). Plozasiran administered as a 25 mg subcutaneous injection once every three months and showed a median 59% reduction in triglycerides levels in clinical trials.^5,6^

 Another siRNA therapeutic is ARO-ANG3 that is under phase 2 clinical trial for management of mixed dyslipidemia that works by inhibiting the hepatic expression of angiopoietin-like protein 3 (ANGPTL3).^7^ Other siRNAs that affect lipoprotein (a) include olpasiran and SLN360 that are in early phase of clinical investigation.^8^

## Cardiac Amyloidosis

 Cardiac amyloidosis is a genetic disorder identified by depositions of transthyretin molecules and accumulation of large amyloid proteins, which is represented by polyneuropathy or autonomic dysfunction followed by fatal cardiomyopathy within 5 to 10 years, if it remains untreated.^1^

 RNA therapy is well documented in the management of cardiac amyloidosis. Vutrisiran, a siRNA that is a derivation of *N*-acetylgalactosamine that inhibits transthyretin (TTR) gene expression and has been approved for the treatment of the polyneuropathy of hereditary transthyretin-mediated (hATTR) amyloidosis in adults.^9^ Vutrisiran is also under phase 3 evaluation for Familial Amyloid Cardiomyopathy in the HELIOS-B trial.^1,9^ Patisiran is another FDA approved siRNA for management of cardiac amyloidosis that degrades mutant and wild-type TTR.^10^

## Other Cardiovascular Disease

 The VEGF-A mRNA AZD8601 is the most advanced therapeutic for revascularization.^1^ An early phase of clinical trial evaluated AZD8601 intradermal injection in human volunteers showed increases in local perfusion.^11^ The EPICCURE trial was a phase 2a trial investigated intracardiac delivery of AZD8601 among patients undergoing coronary artery bypass grafting surgery (CABG) with moderately decreased left ventricular ejection fraction (EF) of 30%–50%.^12^ The trial showed safety and tolerability of AZD8601; however, due to small number of study and technical issues in delivery of mRNA, it could not show a promising result.^12^

 MRG-110 is a class of anti-miRNA that targets anti-microRNA (miR)-92a and promotes revascularization and leads to functional improvements following myocardial infarction which is under primary phase of clinical trials.^1^ CDR132L is another class of anti-miRNA that targets miR-132-3p and reduce cardiac fibrosis.^1^ A phase 1b trial of intravenous CDR132L on 28 chronic heart failure patients with left ventricular EF of ≥ 30% and < 50% or NT-proBNP > 125 ng/L, showed a promising resolution in cardiac fibrosis with a significant QRS narrowing. ^13^

## Future Prospective and Conclusion

 Currently, RNA therapeutics provide a promising perspective in management of CVD. siRNA is the most studied class of RNA based therapies, which has opened a new treatment perspective in management of hypertension, and dyslipidemia, and cardiac amyloidosis and received an FDA label indication. However, larger clinical trials are needed to be proved in the other cardiovascular area such as revascularization, and cardiac regeneration. Based on available trials, the safety and tolerability of RNA therapeutics were desirable. Their limitations in clinical use may include cost-effeteness of therapy, interactions by medications and other therapies, long-term safety profile, and technical issues in synthesis and delivery.

 As a conclusion, RNA therapeutics play as paradigm shifter in the management of CVD, however, larger clinical trial is needed to prove their safety and efficacy in the management of CVD.

## Competing Interests

 The Authors are the Editorial Board of the Journal of Cardiovascular and Thoracic Research at the time of submission. This had no influence on the peer-review process or the final editorial decision.

## Ethical Approval

 Not applicable.
